
JOURNAL INFORMATION
==============================
NLM Title Abbreviation: ScientificWorldJournal
Journal ID: ScientificWorldJournal
NLM Journal ID: 101131163
Journal ID: TSWJ

The Scientific World Journal

ISSN: 2356-6140
EISSN: 1537-744X
Publisher: Wiley

ARTICLE INFORMATION
==============================
PMCID: PMC6009351
PMID: 29973817
DOI: 10.1100/tsw.2002.43
Article ID: 174329
Article version: 1
Subjects: Short Report

Multi-Protein Complexes Studied by Mass Spectrometry

Andersen Jens S. 1
Rappsilber Juri 1
Steen Hanno 1
Pandey Akhilesh 1
Molina Henrik 2
Lund Michael 2
Podtelejnikov Alexandre 2
Lamond Angus I. 3
Mann Matthias 1 2 *
University of Southern Denmark, Dept. Biochemistry and Molecular Biology, Campusvej 55, DK-5230 Odense M, Denmark
MDS-Proteomics, Staermosegaardsvej 6, DK-5230 Odense M, Denmark
Department of Biochemistry, Wellcome Trust Building, University of Dundee, Dundee DD1 4HN, UK
*Matthias Mann: mann@bmb.sdu.dk
Publication date: 2002
Electronic publication date: 2002 Apr 19
Volume: 2
First page: 91
Last page: 92
Copyright: Copyright © 2002 Jens S. Andersen et al.
Copyright year: 2002
License: This is an open access article distributed under the Creative Commons Attribution License, which permits unrestricted use, distribution, and reproduction in any medium, provided the original work is properly cited.
License URL: https://creativecommons.org/licenses/by/3.0/

Funding: Danish Foundation for Basic Research Wellcome Trust

==============================
Advances in Gene Technology: The Genome and Beyond –
Structural Biology for Medicine (Proceedings of the 2002 Miami
*Nature Biotechnology* Winter Symposium)
*TheScientificWorld* 2002, 2(S2), 91–92
ISSN 1532-2246; DOI 10.1100/tsw.2002.43

### MULTI-PROTEIN COMPLEXES STUDIED BY MASS SPECTROMETRY

Jens S. Andersen[1], Juri Rappsilber[1], Hanno Steen[1], Akhilesh Pandey[1], Henrik Molina[2], Michael Lund[2], Alexandre Podtelejnikov[2], Angus I. Lamond[3], Matthias Mann[1,2,]*
[1]University of Southern Denmark, Dept. Biochemistry and Molecular Biology, Campusvej 55, DK-5230 Odense M, Denmark; [2]MDS-Proteomics, Staermosegaardsvej 6, DK-5230 Odense M, Denmark; [3]Department of Biochemistry, Wellcome Trust Building, University of Dundee, Dundee DD1 4HN, U.K.
mann@bmb.sdu.dk

**INTRODUCTION.** Methods of biological mass spectrometry have improved at a dramatic pace ever since the discovery of electrospray ionization (ES) and Matrix assisted laser desorption ionization (MALDI). These methods now allow the relatively rapid identification of gel-separated proteins as well as the analysis of complex protein mixtures. Continuing efforts aim at increasing the sensitivity of mass spectrometric analysis which currently allows peptide sequencing at the low femtomole level, facilitating the sequencing of silver stained proteins and the detection of medium to low abundant proteins in complex mixtures. One of the goals of our groups has been to apply these methods towards the analysis of protein-protein interactions and the molecular elucidation of multiprotein complexes[1,2].

**METHODS.** Gel separated proteins were visualized, excised, and digested as previously described[3] and analyzed by nanoelectrospray sequencing as described[4]. For direct complex mixture analysis, peptides were enzymatically degraded and peptides were separated by HPLC and on-line infused into the mass spectrometer. Mass spectrometric analysis was on PE-Sciex QSTAR Pulsar. Protein databases, EST databases and the draft human genome sequence were searched with the MASCOTT program (Matrix Sciences) and the PepSea software suite (MDS-Proteomics).

**RESULTS.** A large number of multiprotein complexes in mammalian cells have been analyzed by the mass spectrometric methods listed above. These complexes include the spliceosome, the centrosome, mitochondria, and many others. In depth analysis of the nucleolus by a layered approach involving MALDI mass spectrometry and directed sequencing with direct searching of the human draft genome yielded more than 260 proteins. Surprisingly, 30% of these proteins were novel or had no function attached to them (Andersen et al., in press). As in the case of the analysis of the human spliceosome, a number of the novel factors were tagged with the green or yellow fluorescent proteins to determine localization *in vivo*. Based on the results of this experiment, initial florescence and electron microscopy and the identity of the found proteins we estimate the purity of the preparation to be very high. Bioinformatic analysis of the found factors revealed no common targeting signal to the nucleolus. Subsequent analysis of the nucleolus by direct mass spectrometric analysis of the crude mixture served to identify factors which were present or absent in particular cell states such as transcription inhibition.

Mass spectrometry based methods can also be used to study dynamic events such as signaling. Several new members of the EGFR pathway were isolated by antiphosphotyrosine antibodies and some of them are under active study in our laboratory. Novel phosphorylation sites were detected in a number of such factors using a phosphotyrosine specific precursor ion scan[5].

**ACKNOWLEDGEMENT.** Work in MM's laboratory is supported by a generous grant by the Danish Foundation for Basic Research and A.I.L.'s laboratory is supported by the Wellcome Trust.

### REFERENCES

1. Pandey, A. and Mann, M. (2000) Nature 405, 837–846.
2. Lamond, A.I. and Mann, M. (1997) Trends Cell Biol. 7, 139–142.
3. Shevchenko, A., Wilm, M., Vorm, O., and Mann, M. (1996) Anal. Chem. 68, 850–858.
4. Wilm, M., Shevchenko, A., Houthaeve, T., Breit, S., Schweigerer, L., Fotsis, T., and Mann, M. (1996) Nature 379, 466–469.
5. Steen, H., Kuster, B., Fernandez, M., Pandey, A., and Mann, M. (2001) Anal. Chem. 73, 1440–1448.
